# Clinical Competence and Job Market Readiness of Prosthetics and Orthotics Graduates: A Cross-Sectional Study

**DOI:** 10.3390/healthcare14162531

**Published:** 2026-08-13

**Authors:** Mahmoud Alfatafta, Nizar Alsubahi, Huda Alfatafta, Anthony McGarry, Amani Al-Refai, Mohannad Alkhateeb, Noha Alaggad, Alaeddin Ahmad

**Affiliations:** 1 Department of Prosthetics and Orthotics, School of Rehabilitation Sciences, The University of Jordan, Amman 11942, Jordan; 2 Department of Health Service and Hospital Administration, Faculty of Economics and Administration, King Abdulaziz University, Jeddah 21589, Saudi Arabia; 3 Department of Health Services Research, Care and Public Health Research Institute—CAPHRI, Maastricht University Medical Center, Faculty of Health, Medicine and Life Sciences, Maastricht University, P.O. Box 616, 6200 MD Maastricht, The Netherlands; 4 Faculty of Health Sciences, University of Pecs, 7621 Pecs, Hungary; 5 Biomedical Engineering, The University of Strathclyde, Glasgow G4 0NW, UK; 6 Department of Basic Sciences and Humanities, Faculty of Arts and Humanities, Applied Science Private University, Amman 11937, Jordan; 7 School of Public Health and Community Medicine, Institute of Medicine, University of Gothenburg, 40530 Gothenburg, Sweden; 8 Department of Managing Health Services and Hospitals, College of Business, King Abdulaziz University, Rabigh 25726, Saudi Arabia; 9 Department of Marketing, School of Business, The University of Jordan, Amman 11942, Jordan

**Keywords:** clinical competence, job market readiness, prosthetics and orthotics education, graduate employability, openness, structural equation modeling, Jordan

## Abstract

Background: Clinical competence is a fundamental outcome of prosthetics and orthotics (P&O) education and is expected to facilitate graduates’ transition into professional practice. However, limited evidence exists regarding the relationship between clinical competence and job market readiness among P&O graduates, particularly in low- and middle-income countries. Objective: To examine the relationship between perceived clinical competence and job market readiness among prosthetics and orthotics graduates in Jordan and to investigate whether openness moderates this relationship. Methods: A cross-sectional survey was conducted among graduates of the Bachelor of Science in Prosthetics and Orthotics program at the University of Jordan who had graduated within the previous five years. Data were collected between 1 July and 31 August 2025 using a structured online questionnaire comprising measures of clinical competence, job market readiness, and openness. Confirmatory factor analysis (CFA) was performed to assess the reliability and validity of the measurement model, followed by structural equation modeling (SEM) to examine the proposed relationships. The moderating effect of openness was further evaluated using Hayes’ PROCESS macro. Results: A total of 231 questionnaires were included in the final analysis. The measurement model demonstrated satisfactory reliability, convergent validity, discriminant validity, and overall model fit. Clinical competence was a significant positive predictor of job market readiness (β = 0.427, *p* = 0.013), explaining 37% of the variance in job market readiness. Openness did not have a significant direct effect on job market readiness (*p* = 0.072); however, it significantly moderated the relationship between clinical competence and job market readiness (B = 0.295, *p* = 0.021), indicating that the positive association between clinical competence and job market readiness was stronger among graduates with higher levels of openness. Conclusions: Clinical competence plays a central role in enhancing graduates’ readiness for professional employment in prosthetics and orthotics, while openness strengthens this relationship. These findings suggest that prosthetics and orthotics educational programs should integrate strategies that foster both clinical competence and adaptive personal attributes, and they may inform curriculum development and educational policies aimed at improving graduate employability and workforce readiness.

## 1. Introduction

Prosthetics and orthotics (P&O) is a specialized healthcare profession that plays a critical role in restoring mobility, improving functional independence, and enhancing quality of life for individuals with physical impairments and limb loss [1]. P&O professionals are responsible for the assessment, design, fabrication, fitting, and rehabilitation management of individuals requiring prosthetic and orthotic interventions. As healthcare systems continue to evolve and rehabilitation services become increasingly patient-centered, the expectations placed upon newly graduated P&O practitioners have expanded considerably [2]. Graduates are now expected not only to demonstrate technical proficiency and clinical competence, but also to possess adaptability, communication skills, problem-solving abilities, and readiness to function effectively within multidisciplinary healthcare environments [2,3,4,5].

Clinical competence is widely recognized as one of the most fundamental outcomes of healthcare education programs [6]. It encompasses the integration of theoretical knowledge, psychomotor abilities, clinical reasoning, communication skills, and professional behaviors required for safe and effective patient care [3,6]. Within prosthetics and orthotics, clinical competence extends beyond technical fabrication skills to include patient assessment, gait analysis, alignment procedures, follow-up care, outcome evaluation, and the ability to apply evidence-based practice in clinical decision-making [7,8,9]. The increasing incorporation of digital technologies such as CAD/CAM systems, outcome measurement tools, and advanced rehabilitation approaches has further expanded the competencies required from graduates entering the workforce [10].

Despite the importance of clinical competence, many healthcare graduates globally experience difficulties during the transition from academic education to professional employment [11,12]. Several studies have reported concerns regarding graduate preparedness, confidence, employability, and readiness for clinical practice across rehabilitation and allied health professions [13,14]. Job market readiness refers to the extent to which graduates perceive themselves as prepared to meet workplace expectations, adapt to professional demands, and successfully integrate into employment settings [15]. It includes not only technical and practical skills, but also soft skills, adaptability, communication, teamwork, and the ability to respond to workplace challenges effectively [16].

Within the field of prosthetics and orthotics, the issue of graduate readiness is particularly important due to the highly practical and clinically demanding nature of the profession [17]. Newly graduated practitioners are frequently required to independently manage complex patient cases, perform technical fabrication procedures, communicate with multidisciplinary rehabilitation teams, and adapt to rapidly changing clinical technologies [18]. Inadequate clinical competence may negatively influence graduates’ confidence, employability, and ability to transition successfully into the workforce [11]. Consequently, educational institutions are increasingly under pressure to ensure that graduates possess the competencies required for safe clinical practice and sustainable employment outcomes (ISPO, 2024) [19].

In Jordan and many low- and middle-income countries, the demand for qualified prosthetists and orthotists continues to increase because of trauma, diabetes-related amputations, congenital conditions, neurological disorders, and musculoskeletal impairments [20,21]. At the same time, rehabilitation systems in the region continue to face workforce shortages, resource limitations, and variability in clinical training opportunities [22]. These challenges place additional importance on the quality of P&O education and the preparedness of graduates entering the healthcare workforce. Although research examining clinical outcomes and patient satisfaction in prosthetics and orthotics has increased in recent years, there remains limited evidence investigating the clinical competence and job market readiness of P&O graduates, particularly within the Arab region and the Jordanian context.

Moreover, graduate readiness may not depend solely on technical competence. Individual psychological and behavioral characteristics may also influence how graduates adapt to professional environments and employment challenges [23]. One important factor is openness, which refers to an individual’s willingness to learn, adapt, seek new experiences, accept feedback, and engage in continuous professional development [24,25]. In the present study, openness is conceptualized as a professional attribute reflecting adaptability, lifelong learning, and willingness to embrace new knowledge and workplace challenges, rather than the personality trait Openness to Experience described in the Five-Factor Model of personality. This conceptualization aligns with the competencies required for contemporary prosthetics and orthotics practice and the continuous professional development expected of healthcare professionals. In healthcare professions, openness may enhance graduates’ ability to cope with workplace demands, integrate into multidisciplinary teams, and pursue ongoing learning opportunities [26,27]. Graduates who demonstrate higher openness may therefore be better able to translate their clinical competence into successful job market readiness.

Although several personal characteristics, including resilience, self-efficacy, and career adaptability, have been associated with graduate employability [23], the present study focused specifically on openness because it reflects an individual’s willingness to embrace new experiences, acquire new knowledge, accept feedback, and adapt to changing professional environments [28]. These characteristics are particularly relevant to the prosthetics and orthotics profession, where practitioners are expected to engage in lifelong learning, adopt emerging technologies, and respond to rapidly evolving clinical practices [29]. Unlike resilience, which primarily reflects the ability to recover from adversity, or self-efficacy, which reflects confidence in one’s capabilities [30], openness is conceptually aligned with continuous learning and adaptability. Consequently, openness was hypothesized to function as a moderating variable rather than a direct predictor because it was expected to influence how effectively graduates apply and translate their existing clinical competence into job market readiness. Graduates with higher levels of openness are more likely to embrace feedback, engage in lifelong learning, adapt to new clinical environments, and respond to emerging technologies, thereby strengthening the positive influence of clinical competence on professional readiness.

The previous literature has highlighted the importance of adaptability, lifelong learning, and professional flexibility in improving employability outcomes among healthcare graduates [23,31]. However, limited research has explored the moderating role of openness in the relationship between clinical competence and job market readiness, especially within rehabilitation sciences and prosthetics and orthotics education. Understanding this relationship may provide important insights for educational institutions seeking to strengthen graduate preparedness and employability through both technical and professional development strategies.

Therefore, the present study aimed to examine the relationship between perceived clinical competence and job market readiness among prosthetics and orthotics graduates in Jordan and to investigate whether openness moderates this relationship. The findings of this study may contribute to improving curriculum development, clinical education, graduate employability strategies, and workforce preparedness within prosthetics and orthotics education programs in Jordan and similar healthcare contexts.

## 2. Methods

### 2.1. Study Design

This study employed a quantitative cross-sectional research design to examine the relationship between clinical competence and job market readiness among prosthetics and orthotics graduates in Jordan. In addition, the study investigated the moderating role of openness in influencing the relationship between clinical competence and job market readiness. A cross-sectional approach was considered appropriate because it allows the assessment of participants’ perceptions and experiences at a single point in time and is widely used in educational and employability research. The study adopted a hypothesis-testing approach using Structural Equation Modeling (SEM) to evaluate the proposed research model and relationships among the study variables.

### 2.2. Study Population and Sampling

The target population consisted of graduates of the Bachelor of Science in Prosthetics and Orthotics program at the University of Jordan who had graduated within the previous five years. The sampling frame was established using official departmental graduation records and the available alumni contact database. These records were reviewed to identify all graduates who met the eligibility criteria and to remove any duplicate or ineligible entries.

A total population sampling approach was adopted, whereby all 267 eligible graduates identified in the sampling frame were intended to be invited to participate. Of these, 260 graduates were successfully contacted and received the questionnaire. Seven eligible graduates could not be reached because their email messages were returned as undeliverable or no valid contact details were available. This approach was selected to maximize coverage of the accessible graduate population and reduce selection bias.

Although all eligible graduates were invited to participate, a minimum sample size of 200 participants was considered sufficient for Structural Equation Modeling (SEM), as recommended by Hair et al. and Kline, to provide stable parameter estimates and adequate statistical power [32,33].

### 2.3. Instrument Development

The questionnaire was developed for the purposes of this study and was informed by an extensive review of the literature on prosthetics and orthotics education, healthcare professional competence, graduate employability, and lifelong learning. Rather than adopting a single existing instrument, questionnaire items were developed to reflect the conceptual definitions of the study constructs while ensuring relevance to the prosthetics and orthotics profession in Jordan. Clinical competence items were derived from published competency standards and educational frameworks for prosthetists and orthotists, emphasizing patient assessment, prosthetic fabrication, clinical decision-making, gait analysis, communication, and professional practice [14,16,34]. Job market readiness items were informed by the graduate employability literature, particularly studies addressing preparedness for employment, workplace readiness, and professional transition [16,23,35]. The openness construct was developed based on the literature describing adaptability, lifelong learning, receptiveness to feedback, and continuous professional development within healthcare education [26,28].

Content validity was established through expert review prior to data collection. The preliminary questionnaire was independently evaluated by three experts, including two academic staff members in prosthetics and orthotics and one senior clinical prosthetist. The reviewers assessed each item for relevance to the intended construct, clarity of wording, comprehensiveness, and appropriateness for recent graduates of prosthetics and orthotics programs. Based on their feedback, several items underwent minor wording revisions to improve clarity, reduce ambiguity, and ensure that the questionnaire adequately reflected the constructs of perceived clinical competence, job market readiness, and openness. No items were removed or added following the expert review.

Following the expert review, the revised questionnaire was pilot tested with 10 recent prosthetics and orthotics graduates who met the study eligibility criteria but were not included in the final analysis. The pilot study was conducted to assess the clarity, comprehensibility, and feasibility of the questionnaire, as well as the time required for completion. Participants reported that the questionnaire was clear and easy to complete within the estimated timeframe. Minor wording revisions were made based on participant feedback to further improve clarity and readability, while no changes were made to the questionnaire structure or study constructs.

The questionnaire consisted of four sections. The first section collected demographic information, including age, gender, year of graduation, employment status, workplace sector, and years of professional experience.

The second section measured graduates’ perceived clinical competence. This construct was assessed using nine self-reports that evaluated participants’ perceptions of their competence in performing key prosthetics and orthotics clinical tasks, including patient communication and history taking, transtibial prosthetic casting, positive cast rectification, static and dynamic alignment, gait analysis and correction, upper-limb prosthetic fitting, workshop fabrication skills, CAD/CAM utilization, and patient follow-up management.

The third section measured job market readiness using four items assessing participants’ perceptions of preparedness for employment, possession of practical skills required by employers, ability to adapt to workplace demands, and adequacy of educational preparation for employment.

The fourth section assessed openness using five items evaluating willingness to learn new skills, adapt to diverse professional environments, seek professional development opportunities, accept constructive feedback, and relocate or work in different settings to improve employment opportunities. Accordingly, the openness items were designed to measure professional adaptability and lifelong learning behaviours rather than stable personality characteristics, with emphasis on willingness to learn new skills, adapt to different work environments, seek professional development opportunities, accept constructive feedback, and remain flexible in pursuing employment.

All items were measured using a five-point Likert scale ranging from 1 (strongly disagree/very low competence) to 5 (strongly agree/very high competence), with higher scores indicating stronger agreement or higher perceived competence. A copy of the questionnaire is in Supplementary Materials.

### 2.4. Data Collection Procedure

Data were collected using a self-administered online questionnaire hosted on Google Forms. The survey link was distributed through multiple channels, including alumni networks, professional prosthetics and orthotics groups, social media platforms, and direct communication with eligible graduates. Participants were invited to voluntarily complete the questionnaire after reviewing the study information sheet and providing electronic informed consent. The questionnaire required approximately 10–15 min to complete.

Data collection was conducted over an eight-week period from 1 July 2025 to 31 August 2025. During this period, reminder messages were distributed periodically through the same recruitment channels to encourage participation and maximize the response rate.

No research assistants were involved in participant recruitment or data collection. The questionnaire was completed independently by participants through the online platform without any direct interaction with the research team, thereby minimizing the potential for researcher influence on participation or responses.

Ethical approval for this study was obtained from the Institutional Review Board (IRB) of the University of Jordan (Approval No. 444/2025). Participation was voluntary, and electronic informed consent was obtained from all participants prior to accessing the questionnaire. Participants were informed about the purpose of the study, the anonymous nature of data collection, and their right to withdraw from the study at any time without consequence.

No personally identifiable information was collected as part of the survey. Any optional contact information provided by participants for future correspondence was stored separately from survey responses and was not linked to the study data. All study procedures were conducted in accordance with the ethical principles outlined in the Declaration of Helsinki.

### 2.5. Validity and Reliability

Content validity was established through an extensive review of the literature and consultation with experts in prosthetics and orthotics education, clinical practice, and healthcare research. The questionnaire was evaluated for clarity, relevance, and comprehensiveness before data collection.

Construct validity was assessed using Confirmatory Factor Analysis (CFA). Convergent validity was evaluated through standardized factor loadings, Composite Reliability (CR), and Average Variance Extracted (AVE). Following established recommendations, factor loadings greater than 0.50, CR values above 0.70, and AVE values exceeding 0.50 were considered acceptable indicators of convergent validity [32].

Discriminant validity was assessed using the Fornell–Larcker criterion, whereby the square root of the AVE for each construct was compared against its correlations with other constructs. Discriminant validity was considered satisfactory when the square root of AVE exceeded the corresponding inter-construct correlations [36].

Internal consistency reliability was evaluated using Composite Reliability (CR), with values above 0.70 indicating acceptable reliability.

### 2.6. Research Model and Hypotheses

The conceptual framework proposed that clinical competence positively influences job market readiness among prosthetics and orthotics graduates. Furthermore, openness was hypothesized to strengthen this relationship by enhancing graduates’ willingness to learn, adapt, and engage in professional development activities.

Accordingly, the following hypotheses were tested:

**H1.**  *Clinical competence has a significant positive effect on job market readiness among prosthetics and orthotics graduates in Jordan.*

**H2.**  *Openness positively moderates the relationship between clinical competence and job market readiness among prosthetics and orthotics graduates in Jordan.*

### 2.7. Data Analysis

Data were analyzed using the Statistical Package for the Social Sciences (SPSS) version 28 and Analysis of Moment Structures (AMOS) version 26.

Prior to the main analyses, the dataset was screened for completeness and compliance with the assumptions underlying multivariate analysis. Missing data were examined, and questionnaires with incomplete responses that could affect the statistical analyses were excluded from the final dataset. Univariate normality was assessed using skewness and kurtosis statistics, while multicollinearity among the study constructs was evaluated using variance inflation factor (VIF) and tolerance values. Potential multivariate outliers were assessed using Mahalanobis distance. The results indicated no serious violations of normality, multicollinearity, or influential outliers; therefore, the data were considered suitable for confirmatory factor analysis and structural equation modeling.

Confirmatory Factor Analysis (CFA) was conducted to evaluate the measurement model and assess construct validity. Model fit was evaluated using multiple goodness-of-fit indices, including the Comparative Fit Index (CFI), Incremental Fit Index (IFI), Normed Fit Index (NFI), Goodness-of-Fit Index (GFI), Adjusted Goodness-of-Fit Index (AGFI), Root Mean Square Error of Approximation (RMSEA), and Chi-square to degrees of freedom ratio (χ^2^/df). Acceptable model fit was indicated by CFI, IFI, NFI, and GFI values greater than 0.90, RMSEA values below 0.08, and χ^2^/df values below 3.0 [32,33].

Although the questionnaire was newly developed for this study, the measurement model was theory-driven rather than exploratory. The study was based on three predefined latent constructs; perceived clinical competence, job market readiness, and openness. These were identified through the literature review and informed by established competency frameworks and graduate employability theory. Furthermore, the questionnaire underwent expert review to establish content validity prior to data collection. Consequently, confirmatory factor analysis (CFA) was considered the most appropriate approach to evaluate whether the observed data adequately fit the hypothesized measurement model, whereas exploratory factor analysis was not considered necessary because the underlying factor structure had been specified a priori.

Structural equation modeling (SEM) was selected because the study sought to examine relationships among latent constructs measured by multiple observed indicators while simultaneously accounting for measurement error [32,33]. Unlike conventional regression analysis, which assumes that variables are measured without error and typically requires the use of composite scores, SEM enables the measurement model and structural model to be evaluated within a single analytical framework. This approach allowed the reliability and validity of the latent constructs to be established through confirmatory factor analysis prior to testing the hypothesized relationships, thereby providing more robust parameter estimates and greater confidence in the structural findings.

Structural Equation Modeling (SEM) was subsequently performed to test the hypothesized direct relationships among the study constructs. To examine the moderating effect of openness, the product indicator approach was employed. This method creates interaction terms between the indicators of the predictor and moderator constructs and is considered one of the most rigorous approaches for testing latent variable interactions within SEM frameworks [32,37].

To further examine the moderation effect identified in the SEM analysis, Hayes’ PROCESS Macro (Model 1) [38] was subsequently applied using SPSS with observed composite scores. The moderation analysis assessed whether openness significantly influenced the strength of the relationship between clinical competence and job market readiness. Statistical significance was established at a *p*-value of less than 0.05.

The SEM and PROCESS analyses served complementary purposes. SEM was used to estimate the latent variable model while accounting for measurement error and to test the hypothesized structural relationships. PROCESS analysis was subsequently conducted to further examine the interaction effect using observed composite scores and to provide a complementary regression-based assessment of the moderation relationship. Consequently, coefficients of determination (R^2^) derived from the two approaches are not directly comparable because they originate from different analytical models [33,38].

## 3. Results

A total of 267 graduates met the study eligibility criteria and were included in the sampling frame. Of these, 260 were successfully contacted and received the electronic questionnaire, while seven could not be reached because of undeliverable email addresses or unavailable contact information. Overall, 239 responses were received, corresponding to a response rate of 91.9% among successfully contacted graduates and 89.5% of the total eligible population. Following data screening, eight questionnaires were excluded because of incomplete responses or missing data. Consequently, 231 questionnaires were retained for the final analysis, representing 88.8% of the questionnaires distributed, 96.7% of the responses received, and 86.5% of the total eligible population.

Regarding gender distribution, females constituted the majority of the study sample (73%), whereas males accounted for 27% of respondents.

Regarding employment status, 99 participants (41.4%) were unemployed, 89 (37.2%) were employed full-time, and 51 (21.3%) were employed part-time. Thus, although unemployment represented the largest single employment category, more than half of the respondents (58.6%) were employed either full-time or part-time at the time of data collection.

### 3.1. Measurement Model Assessment

The results demonstrated that all item factor loadings exceeded the recommended threshold of 0.70, ranging from 0.722 to 0.830 for clinical competence, 0.825 to 0.862 for job market readiness, and 0.784 to 0.849 for openness (Table 1).

Composite reliability (CR) values were 0.854 for clinical competence, 0.842 for job market readiness, and 0.829 for openness. Average variance extracted (AVE) values were 0.665, 0.643, and 0.611, respectively (Table 1).

Model fit indices indicated an acceptable fit of the measurement model to the data. The CFA results yielded χ^2^ (*p* < 0.001), RMSEA = 0.063, GFI = 0.901, AGFI = 0.829, NFI = 0.904, IFI = 0.915, CFI = 0.915, and χ^2^/df = 2.006 (Table 2).

The mean score for openness was 4.15 (SD = 0.58), followed by perceived clinical competence with a mean of 3.98 (SD = 0.61). Job market readiness had a mean score of 3.72 (SD = 0.69).

Discriminant validity was assessed using the Fornell–Larcker criterion. As shown in Table 3, the square root of the AVE for each construct exceeded its correlations with the other constructs. The square roots of AVE were 0.864 for clinical competence, 0.812 for job market readiness, and 0.806 for openness.

### 3.2. Structural Model Assessment and Hypothesis Testing

The results indicated a significant positive effect of clinical competence on job market readiness (β = 0.427, B = 0.492, SE = 0.041, *p* = 0.013). The model explained 37% of the variance in job market readiness (R^2^ = 0.37). Therefore, H1 was supported (Table 4).

The R^2^ value reported for the direct effect was estimated from the SEM structural model. The R^2^ values reported in the PROCESS moderation analysis represent observed-variable regression models and are not directly comparable with SEM estimates.

The moderating effect of openness was examined using the product indicator approach. Clinical competence remained a significant predictor of job market readiness (B = 0.147, SE = 0.102, t = 2.90, *p* = 0.0035), whereas the direct effect of openness was not statistically significant (B = 0.203, SE = 0.132, t = 1.90, *p* = 0.0721). The interaction term between clinical competence and openness was statistically significant (B = 0.295, SE = 0.109, t = 2.40, *p* = 0.0211), indicating a significant moderation effect (Table 4).

To further probe the significant interaction identified in the SEM analysis, Hayes’ PROCESS analysis was conducted using observed composite scores. The inclusion of the interaction term increased the explained variance in job market readiness from R^2^ = 0.14 to R^2^ = 0.29. The interaction effect remained statistically significant (*p* < 0.05).

Accordingly, H2 was supported.

These R^2^ values were obtained from the PROCESS moderation analysis based on observed composite scores and should not be interpreted as equivalent to the R^2^ reported for the SEM structural model (Table 4), which was estimated using latent variables while accounting for measurement error. Accordingly, the two R^2^ values reflect different analytical approaches and are presented for their respective models rather than for direct comparison.

## 4. Discussion

The present study examined the relationship between clinical competence and job market readiness among prosthetics and orthotics graduates in Jordan and investigated the moderating role of openness in this relationship. The findings demonstrated that the measurement model possessed satisfactory psychometric properties, with acceptable levels of reliability, convergent validity, discriminant validity, and model fit (Table 1, Table 2 and Table 3). More importantly, the structural model revealed that clinical competence was a significant positive predictor of job market readiness, while openness significantly moderated this relationship (Table 4).

It should be noted that clinical competence was assessed through graduates’ self-perceptions rather than objective measures of clinical performance. Therefore, the observed relationship reflects perceived competence and perceived job market readiness. Although self-reported competence may not always correspond to objectively measured competence, it remains an important indicator because graduates’ confidence in their abilities can influence their transition into professional practice, willingness to seek employment, and engagement in clinical responsibilities.

The main finding was that clinical competence had a significant positive effect on job market readiness (β = 0.427, *p* = 0.013), explaining 37% of the variance in job market readiness (Table 4). This indicates that graduates who perceived themselves as more clinically competent were more likely to report being prepared to enter the prosthetics and orthotics workforce. This finding is consistent with [39], who argued that perceived clinical competence in prosthetics and orthotics extends beyond knowledge alone and includes clinical skills, technical skills, interpersonal skills, problem-solving, and clinical judgment. The present finding supports this view by showing that these perceived competencies are not only educational outcomes but are also associated with graduates’ perceived readiness for employment.

This result is also consistent with Ash et al. (2015) [34], who developed competency standards for orthotists/prosthetists in Australia and showed that entry-level practice requires a combination of clinical care, technical ability, professional behavior, evidence-based practice, communication, and lifelong learning. Their study emphasized that recent graduates viewed technical skills as essential to their professional role, while experts emphasized clinical care, evidence-based practice, and professional judgment. The present findings align with both perspectives because the clinical competence construct in this study included technical tasks such as transtibial casting, cast rectification, alignment, fabrication, and CAD/CAM use, as well as clinical tasks such as patient communication, gait analysis, and follow-up care (Table 1).

The association between clinical competence and job market readiness also reflects the broader transition of prosthetics and orthotics from a craft-based profession to a clinically integrated healthcare profession. Boone (2020) [40] described this evolution as a shift from “mechanic to clinician,” where prosthetists and orthotists are increasingly expected to combine mechanical skill, technology use, clinical reasoning, and patient-centered care. Similarly, Kogler and Hovorka (2021) [41] argued that contemporary orthotics and prosthetics education must place greater emphasis on clinical decision-making, evidence-based practice, outcome measurement, digital technologies, and interprofessional collaboration. The present findings support these arguments by demonstrating that graduates’ readiness for the job market is strongly linked to their perceived ability to perform clinically relevant and technically relevant tasks.

Compared with the previous literature, the present study adds empirical evidence to an area where evidence remains limited. Forghany et al. (2018) [29] reported that although it is generally assumed that prosthetic and orthotic service quality depends on practitioner education and training, there is limited direct evidence examining how training influences service delivery or professional outcomes. The present study does not examine service quality directly, but it contributes to this gap by showing that perceived clinical competence is significantly associated with graduates’ readiness to enter professional practice. This provides useful evidence from the graduate perspective and highlights the importance of strengthening clinical competence during undergraduate education.

The findings also agree with Spaulding et al. (2020) [17], who emphasized that prosthetics and orthotics education has moved toward competency-based standards, professional preparedness, structured clinical training, and evidence of student outcomes. Spaulding et al. [17] also noted that graduate and employer feedback can provide valuable information about professional preparedness. The current study extends this argument by quantitatively examining graduate perceptions and showing that clinical competence is a meaningful predictor of job market readiness. This is particularly important because graduate readiness is not only a matter of completing a degree but also reflects whether graduates feel capable of meeting the demands of clinical work after graduation.

An important characteristic of the study population was that 41.4% of graduates were unemployed at the time of data collection. Employment status may have influenced perceptions of both clinical competence and job market readiness, as employed graduates may have had greater opportunities to apply and consolidate their clinical skills, whereas unemployed graduates may have had fewer opportunities to reinforce these competencies in practice. The findings should therefore be interpreted within the employment context of the study population.

The relatively high proportion of unemployed graduates (41.4%) presents an important contrast with the positive association observed between perceived clinical competence and job market readiness (Table 4). This apparent discrepancy suggests that being prepared for employment should not be equated with obtaining employment. Clinical competence may provide graduates with the capabilities required for professional practice, but employment ultimately depends on whether the labour market can absorb those capabilities. Factors such as the availability of P&O positions, distribution of rehabilitation services, employer recruitment requirements, workforce planning, and opportunities for early-career clinical experience may therefore constrain employment independently of graduates’ competence [31,35]. The findings consequently point to a potential education-to-employment gap: strengthening clinical competence remains essential, but educational preparation alone cannot resolve unemployment where appropriate professional opportunities are limited. Importantly, the present study did not directly examine the causes of unemployment; therefore, these structural explanations should be regarded as plausible contextual factors requiring further investigation rather than causal conclusions.

A second important finding was that openness significantly moderated the relationship between clinical competence and job market readiness (B = 0.295, *p* = 0.0211; Table 4). This means that the positive relationship between clinical competence and job market readiness became stronger when graduates reported higher levels of openness. In contrast, openness alone was not a statistically significant direct predictor of job market readiness (*p* = 0.0721; Table 4). This distinction is important. It suggests that openness does not replace perceived clinical competence but rather helps graduates translate their competence into perceived workplace readiness.

Although the additional variance explained by the moderating effect of openness was modest, its practical importance should not be overlooked. Psychological and educational factors often contribute incremental rather than substantial improvements in complex outcomes such as employability [35]. In the present study, openness strengthened the relationship between perceived clinical competence and job market readiness, suggesting that graduates who are more willing to learn, adapt, and embrace new experiences are better positioned to translate their clinical competence into successful workforce transition. From an educational perspective, this finding supports the integration of lifelong learning, reflective practice, adaptability, and professional development opportunities within prosthetics and orthotics curricula.

This finding is consistent with the graduate employability literature, which suggests that employability depends not only on discipline-specific skills but also on personal attributes, adaptability, career management, and the ability to respond to changing workplace expectations. Tomlinson (2017) [23] argued that graduate employability is shaped by multiple forms of graduate capital, including human capital, social capital, cultural capital, psychological capital, and identity capital. Similarly, Jackson (2015) [35] emphasized the importance of employability skill development through work-integrated learning, particularly because graduates need to apply their skills in real workplace contexts. The present findings support these perspectives by showing that clinical competence is central, but its effect on readiness is strengthened when graduates are open to learning, feedback, adaptation, and professional development.

The moderation result is also highly relevant to prosthetics and orthotics because the profession is undergoing rapid technological and clinical change. Kogler and Hovorka (2021) [41] argued that future orthotics and prosthetics professionals must adapt to digital shape capture, 3D modeling, additive manufacturing, data-driven decision-making, and evidence-based clinical practice. Spaulding et al. (2020) [17] similarly emphasized lifelong learning, critical inquiry, technology integration, interprofessional collaboration, and professional preparedness as key priorities for future prosthetics and orthotics education. The current findings support these recommendations by showing that openness strengthens the relationship between competence and readiness. Graduates who are clinically competent but less open may be less prepared to adapt those competencies to new technologies, changing workplace environments, or unfamiliar clinical demands.

The findings have important implications for prosthetics and orthotics education in Jordan. The strong relationship between clinical competence and job market readiness suggests that undergraduate programs should continue to prioritize hands-on clinical training, structured patient exposure, technical fabrication skills, gait analysis, alignment, communication, follow-up care, and digital technology training. At the same time, the significant moderation effect of openness suggests that curricula should not focus only on technical and clinical content. Educational programs should also intentionally develop adaptability, reflective practice, feedback-seeking behavior, lifelong learning, and professional flexibility.

International comparisons suggest that the competencies associated with professional readiness are broadly similar across prosthetics and orthotics education systems, although their implementation differs according to national regulation, healthcare organization, and labour-market conditions. Competency frameworks developed in Sweden and Australia emphasize clinical care, technical proficiency, evidence-based practice, communication, professional conduct, and lifelong learning as essential entry-level outcomes [37,38]. Similarly, international reviews of P&O education have highlighted the importance of structured clinical placements, clearly defined competency standards, employer engagement, and continuing professional development [17,29]. The present findings therefore show considerable alignment with international expectations regarding what P&O graduates should be able to do. However, the 41.4% unemployment observed in the present sample suggests that alignment with professional competencies does not necessarily translate into equivalent employment opportunities. This distinction is particularly important when comparing educational systems internationally: competency frameworks primarily define preparedness for professional practice, whereas successful workforce entry also depends on country-specific service structures, professional regulation, workforce demand, and availability of entry-level positions. The Jordanian findings therefore extend the international literature by highlighting the need to consider not only whether graduates acquire appropriate competencies, but also whether the healthcare system provides sufficient pathways through which those competencies can be utilized.

The coexistence of relatively high perceived preparedness with substantial graduate unemployment also has implications beyond university curricula. Addressing this mismatch requires coordination between educational and health-workforce policy. Universities, professional bodies, healthcare providers, and government agencies should collaborate to align graduate numbers and competencies with national rehabilitation workforce needs. Potential strategies include routine graduate and employer surveys, formal advisory committees involving employers, expansion of structured internships and transition-to-practice programmes, clearer competency-based assessment at graduation, and improved workforce data regarding service demand and vacancies. Educational policies should also strengthen exposure to digital technologies, interprofessional practice, career preparation, and service models relevant to resource-constrained settings. Such measures may help reduce the mismatch between educational preparation and labour-market opportunities while ensuring that curriculum development remains responsive to national healthcare priorities.

These implications are particularly relevant in Jordan and similar low- and middle-income contexts, where graduates may enter clinical environments with variable resources, limited access to advanced technologies, and inconsistent clinical placement opportunities. Spaulding et al. (2020) [17] noted that graduates in low- and middle-income countries may face difficulties related to infrastructure, service awareness, and limited resources. Forghany et al. (2018) [29] also highlighted the need to consider education and training in relation to service delivery, particularly in settings where rehabilitation needs are increasing and resources may be constrained. In such contexts, clinical competence is essential, but adaptability and openness may help graduates respond more effectively to workplace limitations and evolving service demands.

Overall, the findings support the argument that job market readiness in prosthetics and orthotics is shaped by both professional competence and individual adaptability. Clinical competence provides the core foundation for practice, while openness appears to enhance the ability of graduates to apply that competence in real employment contexts. Therefore, improving graduate readiness requires more than increasing theoretical knowledge or technical exposure alone. It requires an integrated educational approach that combines clinical competence, practical experience, professional development, adaptability, and lifelong learning.

Several limitations should be considered when interpreting the findings of this study. First, this study assessed graduates’ perceived clinical competence using self-reported measures rather than objective assessments of clinical performance. Consequently, the findings may be influenced by social desirability bias, recall bias, and individual differences in self-perception. Although perceived competence provides valuable insight into graduates’ confidence and preparedness for practice, it may not fully reflect actual clinical competence. Future studies should incorporate objective measures, such as Objective Structured Clinical Examinations (OSCEs), clinical competency assessments, employer evaluations, or supervisor ratings, to complement graduates’ self-reported perceptions. Second, the study relied on self-reported measures, which may be influenced by social desirability bias and participants’ subjective perceptions. Third, the sample was restricted to graduates of a single prosthetics and orthotics program in Jordan, which may limit the generalizability of the findings to graduates from other institutions or countries. Institutional differences in curricula, clinical training opportunities, educational resources, and labour market conditions may influence graduates’ perceptions of clinical competence and job market readiness. Therefore, caution should be exercised when generalizing these findings beyond the University of Jordan, and future multi-institutional studies are warranted to confirm the applicability of the findings across different educational and healthcare settings. Furthermore, because participants differed in their employment status, opportunities to apply clinical skills in practice may have varied and influenced their self-perceptions of competence and job market readiness.

## 5. Conclusions

This study demonstrated that clinical competence is a significant predictor of job market readiness among prosthetics and orthotics graduates in Jordan. In addition, openness was found to strengthen the relationship between clinical competence and job market readiness, highlighting the importance of adaptability and lifelong learning in facilitating the transition from education to professional practice. These findings suggest that prosthetics and orthotics education programs should integrate the development of both technical and clinical competencies with personal attributes such as openness, adaptability, and lifelong learning. Such an integrated educational approach may inform curriculum development and educational policy while enhancing graduate preparedness, employability, and workforce readiness within the prosthetics and orthotics profession.

## Figures and Tables

**Table 1:** Measurement model results, including standardized factor loadings, composite reliability (CR), and average variance extracted (AVE) for clinical competence, job market readiness, and openness.

Questions	Factor Loading	CR	AVE
Clinical competence			
Q1	0.725	0.854	0.665
Q2	0.812		
Q3	0.795		
Q4	0.810		
Q5	0.722		
Q6	0.821		
Q7	0.812		
Q8	0.815		
Q9	0.830		
Job market readiness			
Q1	0.825	0.842	0.643
Q2	0.852		
Q3	0.835		
Q4	0.862		
Openness			
Q1	0.788	0.829	0.611
Q2	0.817		
Q3	0.784		
Q4	0.822		
Q5	0.849		

**Table 2:** Goodness-of-fit indices for the measurement model.

Indices	Value
χ^2^	*p* < 0.001
RMSEA	0.063
GFI	0.901
AGFI	0.829
NFI	0.904
IFI	0.915
CFI	0.915
Normed χ^2^	2.006

χ^2^: Chi-square statistic, RMSEA: Root Mean Square Error of Approximation, GFI: Goodness-of-Fit Index, AGFI: Adjusted Goodness-of-Fit Index, NFI: Normed Fit Index, IFI: Incremental Fit Index, CFI: Comparative Fit Index and Normed χ^2^: Chi-square/Degrees of Freedom (χ^2^/df).

**Table 3:** Discriminant validity of the study constructs based on the Fornell–Larcker criterion.

Variable	Clinical Competence	Job Market Readiness	Openness
Clinical competence	**0.864**		
Job market readiness	0.425	**0.812**	
Openness	0.432	0.429	**0.806**

Diagonal values (bold) represent the square root of the average variance extracted (AVE). Off-diagonal values represent inter-construct correlations.

**Table 4:** Structural model results examining the direct effect of clinical competence on job market readiness and the moderating effect of openness among prosthetics and orthotics graduates in Jordan.

Direct Effect	B	S.E.	β	*p*-Value	R^2^	Hypothesis
CC → JMR	0.492	0.041	0.427	0.013	0.37	Accepted
**Moderation effect**						
**Path**	**Coefficient**	**S.E.**	**T. Value**	**Significance Level**	**LLCI**	**ULCI**
CC → JMR	0.147	0.102	2.90	0.0035	0.051	0.244
O → JMR	0.203	0.132	1.90	0.0721	−0.025	0.421
Interaction effects through moderator	0.295	0.109	2.40	0.0211	0.035	0.652

B = unstandardized regression coefficient; β = standardized regression coefficient; S.E. = standard error; LLCI = lower limit of the 95% confidence interval; ULCI = upper limit of the 95% confidence interval; R^2^ = coefficient of determination. The interaction term represents the moderating effect of openness on the relationship between clinical competence and job market readiness.

## Data Availability

The data underlying this article are available in the article. The datasets generated and analyzed during the current study are available from the corresponding author upon reasonable request. The data is not publicly available due to ethical and privacy restrictions associated with research involving human participants.

## Supplementary Materials

The following supporting information can be downloaded at: https://www.mdpi.com/article/10.3390/healthcare14162531/s1.
