# Supplementary material for: Clinical Competence and Job Market Readiness of Prosthetics and Orthotics Graduates: A Cross-Sectional Study
Source: Healthcare (Basel). 2026 Aug 13;14(16):2531. doi: 10.3390/healthcare14162531 (PMC13512362; doi:10.3390/healthcare14162531)
Supplement: Supplementary file 1 [file healthcare-14-02531-s001.zip › healthcare-4452192-supplementary.pdf]

Number	Question
**Clinical competence**	
Q1	Patient communication and history taking
Q2	Casting for a transtibial prosthesis
Q3	Positive cast rectification
Q4	Static and dynamic alignment
Q5	Gait analysis and correction
Q6	Fitting upper limb prostheses
Q7	Fabrication using basic workshop tools
Q8	Using CAD/CAM tools or software
Q9	Managing patient follow-up and adjustments
**Job market readiness**	
Q1	I feel prepared to enter the job market as a prosthetics and orthotics professional.
Q2	I possess the practical skills required by employers in the prosthetics and orthotics field.
Q3	I am confident in my ability to adapt to workplace demands and challenges.
Q4	My education has equipped me with both technical and soft skills needed for employment.
**Openness**	
Q1	I am open to learning new skills required to improve my employability in the prosthetics and orthotics field.
Q2	I am willing to adapt to different work environments and job roles within my profession.
Q3	I actively seek opportunities (e.g., workshops, training, internships) to enhance my job readiness.
Q4	I am open to feedback and constructive criticism to improve my clinical and professional performance.
Q5	I am willing to relocate or work in different settings to gain employment in my field.
